# Identification and Small Molecule Inhibition of an Activating Transcription Factor 4 (ATF4)-dependent Pathway to Age-related Skeletal Muscle Weakness and Atrophy*[Fn FN2]

**DOI:** 10.1074/jbc.M115.681445

**Published:** 2015-09-03

**Authors:** Scott M. Ebert, Michael C. Dyle, Steven A. Bullard, Jason M. Dierdorff, Daryl J. Murry, Daniel K. Fox, Kale S. Bongers, Vitor A. Lira, David K. Meyerholz, John J. Talley, Christopher M. Adams

**Affiliations:** From the Departments of ‡ Internal Medicine,; ** Molecular Physiology and Biophysics,; §§ Health and Human Physiology, and; ¶¶ Pathology,; the § Fraternal Order of Eagles Diabetes Research Center, and; the ‡‡ College of Pharmacy, The University of Iowa, Iowa City, Iowa 52242,; the ¶ Iowa City Veterans Affairs Medical Center, Iowa City, Iowa 52246, and; ‖ Emmyon, Inc., Coralville, Iowa 52241

**Keywords:** aging, muscle atrophy, protein synthesis, skeletal muscle, skeletal muscle metabolism, ATF4, sarcopenia, skeletal muscle atrophy, tomatidine, ursolic acid

## Abstract

Aging reduces skeletal muscle mass and strength, but the underlying molecular mechanisms remain elusive. Here, we used mouse models to investigate molecular mechanisms of age-related skeletal muscle weakness and atrophy as well as new potential interventions for these conditions. We identified two small molecules that significantly reduce age-related deficits in skeletal muscle strength, quality, and mass: ursolic acid (a pentacyclic triterpenoid found in apples) and tomatidine (a steroidal alkaloid derived from green tomatoes). Because small molecule inhibitors can sometimes provide mechanistic insight into disease processes, we used ursolic acid and tomatidine to investigate the pathogenesis of age-related muscle weakness and atrophy. We found that ursolic acid and tomatidine generate hundreds of small positive and negative changes in mRNA levels in aged skeletal muscle, and the mRNA expression signatures of the two compounds are remarkably similar. Interestingly, a subset of the mRNAs repressed by ursolic acid and tomatidine in aged muscle are positively regulated by activating transcription factor 4 (ATF4). Based on this finding, we investigated ATF4 as a potential mediator of age-related muscle weakness and atrophy. We found that a targeted reduction in skeletal muscle ATF4 expression reduces age-related deficits in skeletal muscle strength, quality, and mass, similar to ursolic acid and tomatidine. These results elucidate ATF4 as a critical mediator of age-related muscle weakness and atrophy. In addition, these results identify ursolic acid and tomatidine as potential agents and/or lead compounds for reducing ATF4 activity, weakness, and atrophy in aged skeletal muscle.

## Teaser

**Background:** Aging reduces skeletal muscle strength and mass.

**Results:** The transcription factor ATF4 is required for age-related muscle weakness and atrophy, and the small molecules ursolic acid and tomatidine reduce ATF4 activity, weakness, and atrophy in aged skeletal muscle.

**Conclusion:** ATF4 is an essential mediator of muscle aging.

**Significance:** These results identify new strategies for reducing weakness and muscle loss during aging.

## Introduction

Skeletal muscle weakness and atrophy are among the most pervasive and disruptive effects of aging. In nearly all people, even elite athletes, a subtle loss of muscle strength begins between the ages of 30 and 40. Over the next two to three decades, strength continues to erode, whereas muscle mass typically declines to a lesser degree. As a result, reduced muscle quality (*i.e.* strength per unit muscle mass) is a hallmark of the aging process (1, 2). By the age of 65, overt muscle loss (age-related muscle atrophy or sarcopenia) is apparent in many individuals, and nearly all elderly persons report a gradual loss of strength and muscle over the course of their lives. The clinical consequences of age-related weakness and muscle loss are significant. Weakness limits activity, impairs quality of life, contributes to falls and fractures, and can create a vicious cycle of muscle disuse and further muscle loss and weakness. In its later stages, age-related muscle atrophy can lead to frailty, debilitation, and loss of independent living. All of these issues are becoming more prevalent as the elderly population increases. For example, in the United States, the number of persons over 65 years old is predicted to more than double between 2010 and 2040 (3).

Despite their broad impact, age-related muscle weakness and atrophy cannot be reliably prevented by physical therapy or current nutritional approaches, and a pharmacologic therapy does not exist. The development of effective interventions has been somewhat hindered by the fact that the molecular basis of age-related muscle weakness and atrophy is largely unknown. The slow progression of age-related skeletal muscle atrophy represents a significant barrier to its experimental study and suggests that the condition may reflect subtle molecular changes that accumulate in skeletal muscle over many years. At the cellular level, age-related muscle atrophy shares some features with acute forms of muscle atrophy caused by fasting, muscle disuse, or systemic illness, which reduce muscle mass and strength over the course of days or weeks rather than years. For example, aging, fasting, muscle disuse, and systemic illness all cause a reduction in skeletal muscle fiber size and a loss of skeletal muscle protein. However, it is also clear that age-related muscle atrophy differs from acute muscle atrophy in some important ways. For example, at least some mediators of acute muscle atrophy (*e.g.* MAFbx/atrogin-1, MuRF1, and AMP kinase) also appear to protect muscle from effects of aging, and thus, chronic deficiencies of those proteins reduce muscle atrophy during acute stress conditions but accelerate the loss of muscle mass and/or quality during aging (4–7). A specific protein target for reducing the loss of muscle quality, strength, and mass during aging has not yet been found.

In the current study, we investigated mechanisms of skeletal muscle weakness and atrophy during aging as well as potential interventions for these conditions. The potential interventions we investigated were two structurally dissimilar small molecules, ursolic acid and tomatidine. Ursolic acid is a naturally occurring pentacyclic triterpene acid present in several edible herbs and fruits, including apples (8). Tomatidine is a naturally occurring steroidal alkaloid derived from tomato plants and green tomatoes (9). We previously found that ursolic acid and tomatidine reduce acute skeletal muscle atrophy caused by fasting and muscle disuse in young adult mice (10–12). In addition, we found that ursolic acid and tomatidine increase muscle strength and quality, and they stimulate muscle hypertrophy when they are administered to healthy young adult mice (10, 11). The mechanisms of action of ursolic acid and tomatidine in skeletal muscle are not well understood; however, both compounds stimulate protein synthesis, protein accretion, and cellular hypertrophy in cultured skeletal myotubes, indicating a direct effect on skeletal muscle cells (10, 11, 13). The effects of ursolic acid and tomatidine on age-related muscle weakness and atrophy were not known. However, despite the existence of mechanistic differences between acute and age-related muscle atrophy, we hypothesized that acute and age-related muscle atrophy might share some common molecular mediators, and thus, age-related muscle weakness and/or atrophy might be reduced by ursolic acid and/or tomatidine.

## Experimental Procedures

### 

#### 

##### Chemicals and Antibodies

We obtained ursolic acid (BML-CT105) and tomatidine (BML-GR335) from Enzo Life Sciences. Puromycin (P8833) was obtained from Sigma-Aldrich. [^3^H]Leucine (120 Ci/mmol) was obtained from American Radiolabeled Chemicals. Anti-myosin heavy chain I (BA-F8), anti-myosin heavy chain IIa (SC-71), anti-myosin heavy chain IIb (BF-F3), and anti-myosin heavy chain IIx (6H1) mouse monoclonal antibodies were obtained from the University of Iowa Developmental Studies Hybridoma Bank. Alexa Fluor 488-conjugated anti-mouse IgG_1_, Alexa Fluor 350-conjugated anti-mouse IgG_2b_, and Alexa Fluor 555-conjugated anti-mouse IgM were obtained from Life Technologies. Anti-FLAG mouse monoclonal antibody (F1804) was obtained from Sigma-Aldrich. Anti-puromycin mouse monoclonal antibody (MABE343) was obtained from EMD Millipore. All other antibodies were obtained from Cell Signaling Technology; these included anti-S6K (catalog number 2308S), anti-phospho-S6K (Thr-389) (catalog number 9234S), anti-Akt (catalog number 4691S), anti-phospho-Akt (Ser-473) (catalog number 4060S), anti-eukaryotic translational initiation factor 4E-binding protein 1 (4E-BP1) (catalog number 9644S), anti-phospho-4E-BP1 (Thr-37/46) (catalog number 2855S), and horseradish peroxidase (HRP)-conjugated anti-rabbit IgG (catalog number 7074S) and anti-mouse IgG (catalog number 7076S).

##### Plasmids and Recombinant Adenoviruses

p-ATF4-FLAG encodes wild-type mouse activating transcription factor 4 (ATF4)^2^ (NM_009716) with three copies of the FLAG epitope tag at the NH_2_ terminus under the control of the CMV promoter. Recombinant adenoviruses expressing GFP alone,GFP plus FLAG-tagged ATF4, or GFP plus FLAG-tagged ATF4ΔbZIP were described previously (14). ATF4ΔbZIP (15) is a full-length ATF4 construct containing three copies of the FLAG epitope tag at the NH_2_ terminus as well as ^292^GLYEAAA^298^ substituted for ^292^RYRQKKR^298^.

##### Mouse Protocols

Animals were housed in colony cages in a specific pathogen-free animal facility at 21 °C with 12-h light/12-h dark cycles and were maintained on standard chow (Harlan Teklad formula 7013) unless otherwise indicated. In all studies, investigators who obtained the results were blinded to the intervention (dietary or genetic). Ursolic acid or tomatidine was added to standard chow formula 7013 at concentrations of 0.27 (w/w) and 0.05% (w/w), respectively, by Harlan Teklad, as described previously (10, 11). 22-month-old male C57BL/6 mice were obtained from the National Institute on Aging and randomly assigned to standard chow diets lacking or containing ursolic acid or tomatidine. Forelimb grip strength was determined using a triangular pull bar attached to a grip strength meter (Columbus Instruments) as described previously (10). *Ex vivo* studies of skeletal muscle force generation were performed as described previously (11). For skeletal muscle transfection experiments, male C57BL/6 mice were obtained from the National Cancer Institute (NCI) at ages 6–8 weeks old and used for experiments within 2 weeks of their arrival. Transfection of mouse skeletal muscle with plasmid DNA was performed as described previously (15). In the SUnSET assay (16), mice were administered an intraperitoneal injection of puromycin (0.04 μmol/g of body weight, dissolved in 100 μl of sterile saline), and then 30 min later, mice were euthanized, and skeletal muscles were harvested and frozen in liquid N_2_ for further analysis. Muscle-specific *Atf4* knock-out (ATF4 mKO) mice were generated and genotyped as described previously (14). Male ATF4 mKO mice (*i.e. ATF4*(*L*/*L*);*MCK-Cre*(*Tg*/*0*) mice) were studied at 22 months of age and compared with male *ATF4*(*L*/*L*);*MCK-Cre*(*0*/*0*) littermates. All animal procedures were approved by the Institutional Animal Care and Use Committee of the University of Iowa.

##### Histology

Skeletal muscle H&E stain and fiber type-specific stain were performed as described previously (11). All histological sections were examined and photographed using an Olympus BX-61 automated upright microscope equipped with CellSens digital imaging software. Image analysis was performed using ImageJ software. Skeletal muscle fiber size was analyzed by measuring the lesser diameter (minimal Feret diameter) of muscle fibers as recommended elsewhere (17).

##### Quantification of Ursolic Acid and Tomatidine in Mouse Plasma

Mouse plasma was collected as described previously (10), and plasma levels of ursolic acid and tomatidine were determined with validated liquid chromatography/mass spectrometry (LC/MS) assays. In the ursolic acid assay, betulinic acid was used as the internal standard. Stock solutions of ursolic acid and betulinic acid were generated by dissolving the compounds in 100% methanol at a concentration of 200 μg/ml, and working solutions were prepared by serial dilution of the stock solutions in 100% methanol. Standard curve samples were generated by adding ursolic acid to 100 μl of blank mouse plasma at concentrations ranging from 1 to 1000 ng/ml. Control samples contained 0, 10, 100, or 700 ng/ml ursolic acid in 100 μl of blank mouse plasma. Betulinic acid (5 μl of a 10 μg/ml stock) was added to the standard curve samples, control samples, and experimental samples (100 μl of plasma from mice treated with control diet, ursolic acid diet, or tomatidine diet). Ursolic acid and betulinic acid were then extracted from plasma samples as follows. 600 μl acetonitrile was added to each sample, and the samples were vortexed for 1 min and allowed to sit for 5 min. 400 μl of ethyl acetate was added to each sample, and the samples were vortexed, centrifuged for 3 min at 14,000 rpm, and then transferred to a Phree Phospholipid Removal plate (product number 8E-S133-TGB, Phenomenex, Torrance CA). A vacuum was applied, and the eluent was collected, transferred to a 13 × 100-mm glass tube, and dried under nitrogen. The dried samples were reconstituted in 100 μl of mobile phase (5 mm ammonium acetate in ultrapure water (15%) and methanol (85%)) and then transferred to HPLC injection vials. LC/MS was performed with a Waters Acquity UPLC system equipped with binary solvent manager, sample manager, and column manager operating under Empower software (Waters Corp., Milford, MA) and a Shimadzu 2010A LC/MS platform in atmospheric pressure chemical ionization negative mode operating under LC/MS Solutions software (version 3.41.324; Shimadzu, Columbia, MD). The analytical column was an Acquity UPLC high strength silica column (C_18_, 1.8 μm, 2.1 × 100 mm; Waters Corp.) with a Phenomenex C_18_ ULTRA UHPLC SecurityGuard column. Separation conditions were as follows: sample temperature, 7 °C; column temperature, 40 °C; sample was injected in partial loop with overfill injection mode; injection volume, 15 μl. The analysis was isocratic at 0.4 ml/min flow. Solvent A (15%) was 5 mm ammonium acetate; solvent B (85%) was methanol. The total run time for an LC/MS analysis was 7 min. The scan interval was 0.3 s with the following parameters: microscan, 0.1 atomic mass unit; atmospheric pressure chemical ionization temperature, 450 °C; curved desolvation line temperature, 200 °C; heat block temperature, 200 °C; nitrogen flow rate, 2.5 liters/min; detector voltage, 1.6 kV. The monitored mass-to-charge ratio for both ursolic acid and betulinic acid was 455.45. The retention times for ursolic acid and betulinic acid were 3.85 and 3.25 min, respectively. The assay was linear over the concentration range of 1–1000 ng/ml, and the coefficient of variation was less than 15% at all control concentrations. In the tomatidine assay, solanidine was utilized as the internal standard. Stock solutions of tomatidine and solanidine were generated by dissolving the compounds in 100% methanol, and working solutions were prepared by serial dilution of the stock solutions in 100% methanol. Standard curve samples were generated by adding tomatidine to 100 μl of blank mouse plasma at concentrations ranging from 4 to 1000 ng/ml. Control samples contained 0, 10, 100, or 700 ng/ml tomatidine in 100 μl of blank mouse plasma. Solanidine (5 μl of a 10 μg/ml stock) was added to the standard curve samples, control samples, and experimental samples (100 μl of plasma from mice treated with control diet, ursolic acid diet, or tomatidine diet). Tomatidine and solanidine were then extracted from plasma samples as follows. Samples were applied to Isolute SLE+ cartridges (6 ml; Biotage, AB, Uppsala, Sweden) in a Cerex solid phase extraction processor (Varian, Palo Alto, CA) and allowed to adsorb onto the packing for 10 min. Samples were eluted with 5 ml of hexanes:dichloromethane (2:1 volume ratio) under gravity flow for ∼15 min. Eluates were collected in 13 × 100-mm glass tubes and evaporated under flowing nitrogen at 25 °C. Dried samples were reconstituted in 200 μl of water containing 40% methanol and 0.05% acetic acid and then transferred to HPLC injection vials. LC/MS was performed with a Shimadzu LC/MS-2010A mass spectrometer operated using electrospray interface in positive ion mode controlled using LC/MS Solutions software (version 3.41.324). The block and curved desolvation line temperature were both set at 250 °C. Data were collected in the selected ion monitoring mode at 416.45 (tomatidine) and 398.45 atomic mass units (solanidine). The analytical column was a Phenomenex Kinetex C_18_ (100 × 2.1 mm, 2.6 μm) preceded by a Phenomenex C_18_ ULTRA UHPLC SecurityGuard column. Separation conditions were as follows: sample temperature, 24 °C (+3 °C); column temperature, 40 °C; sample injection volume, 10 μl. A gradient of 0.25 ml/min was used for the analysis. Solvent A was water with 0.05% acetic acid and 5 mm ammonium acetate, and solvent B was methanol (75%) mixed with acetonitrile (25%). The gradient started at 40% B and increased to 90% B at 5 min, was held for 3 min, and then was changed back to baseline at 9 min. The total run time for LC/MS analysis was 12 min. The retention times for tomatidine and solanidine were 5.4 and 4.6 min, respectively. The assay was linear from 4 to 1000 ng/ml, and the coefficient of variation for all control samples was less than 15%.

##### Immunoblot Analyses

Protein extracts from mouse skeletal muscle and cultured myotubes were prepared as described previously (14). An aliquot of each protein extract was then mixed with 0.25 volume of sample buffer (14) and heated for 5 min at 95 °C. A separate aliquot of each extract was used to determine protein concentration by the BCA kit after which an equivalent amount of protein from each sample was subjected to SDS-PAGE and then transferred to 0.45-μm nitrocellulose membranes (Bio-Rad catalog number 162-0115). Immunoblots were performed at 4 °C for 16 h using primary antibodies diluted to 1:1000 (phospho-S6K), 1:2000 (S6K, Akt, phospho-Akt, and phospho-4E-BP1), 1:3000 (4E-BP1), 1:4000 (puromycin), or 1:5000 (FLAG). Bound antibodies were visualized by chemiluminescence (SuperSignal West Pico, Thermo Scientific) using a 1:2000–1:5000 dilution of HRP-conjugated anti-rabbit or anti-mouse IgG. Membranes were stained with Ponceau S to ensure equal loading.

##### mRNA Expression Analyses

For microarray analyses, skeletal muscle RNA was extracted with TRIzol (Invitrogen) and purified with an RNeasy kit and RNase-free DNase set (Qiagen). RNA was processed and hybridized to Mouse Ref-8 version 2.0 BeadChip arrays (Illumina) by the Southern California Genotyping Consortium (University of California, Los Angeles) as described previously (18). Following hybridization, arrays were washed, blocked, stained, and dried (Little Dipper processor). Arrays were scanned with an iScan reader, and data were extracted and analyzed with BeadStudio software (Illumina). Quantitative real time RT-PCR (qPCR) was performed using TaqMan Gene Expression Assays (Applied Biosystems) and methods described previously (14).

##### Myotube Experiments

Mouse C2C12 myoblasts were obtained from ATCC (CRL-1772) and maintained at 37 °C and 5% CO_2_ in Dulbecco's modified Eagle's medium (DMEM) (ATCC catalog number 30-2002) containing antibiotics (100 units/ml penicillin and 100 μg/ml streptomycin sulfate) and 10% (v/v) fetal bovine serum (FBS). Myoblasts were set up for experiments on day 0 in 6-well plates at a density of 2.5 × 10^5^ cells/well. On day 2, differentiation was induced by replacing 10% FBS with 2% horse serum. On day 7, cells were rinsed once with PBS, and then 1 ml of DMEM containing adenovirus (multiplicity of infection, 250) was added to each well. Two hours later, 1 ml of DMEM containing 1% horse serum plus antibiotics was added to each well. On day 8, cells were rinsed twice with PBS, and then 2 ml of DMEM containing 2% horse serum and antibiotics was added to each well. Infection efficiency was >90%. On day 9 (48 h postinfection), myotubes were harvested for analysis of *Eif4ebp1* and 4E-BP1 expression or used for analysis of global protein synthesis. For analysis of protein synthesis, [^3^H]leucine incorporation was determined as described previously (14).

##### Statistical Analysis

Results of experiments are shown as mean ± S.E. Statistical significance was determined using *t* tests, one-way ANOVA with Dunnett's multiple comparison test, or false discovery rate (FDR) analysis as noted in the figure legends.

## Results

### 

#### 

##### Ursolic Acid and Tomatidine Reduce Age-related Skeletal Muscle Weakness and Atrophy

**FIGURE 1.**
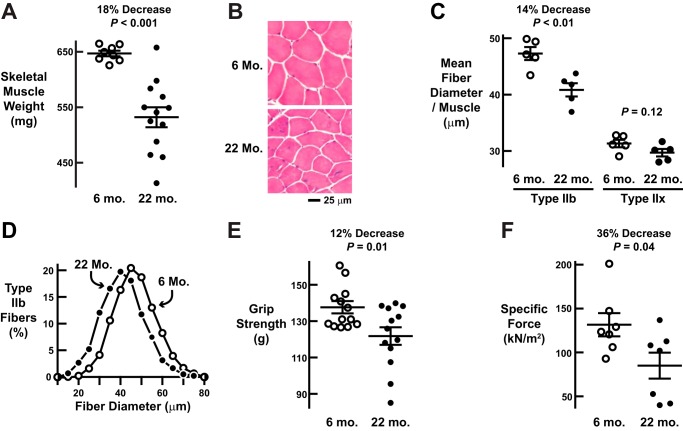
**Mouse model of age-related skeletal muscle weakness and atrophy.** *A–F*, we compared weight-matched cohorts of mature adult (6-month (*mo.*)) and old (22-month) male C57BL/6 mice. *A*, combined weight of bilateral quadriceps femoris and triceps brachii muscles. Each data point represents one mouse, and *horizontal bars* denote means ± S.E. *B*, representative H&E images of quadriceps cross-sections. *C*, average diameters of type IIb and IIx fibers in the quadriceps. Each data point represents the mean of ≥800 type IIb fibers or ≥100 type IIx fibers from one mouse, and *horizontal bars* denote average of the means ± S.E. *D*, size distribution of all type IIb fibers from *C. E*, *in vivo* forelimb grip strength. Each data point represents the mean of five measurements from one mouse, and *horizontal bars* denote the average of the means ± S.E. *F*, *ex vivo* specific tetanic force generated by the extensor digitorum longus muscle. Each data point represents one mouse, and *horizontal bars* denote means ± S.E. In *A*, *C*, *E*, and *F*, *p* values were determined with *t* tests. *kN*, kilonewtons.

Mice have been reported to develop age-related muscle atrophy by the age of 22 months (2, 5, 19). To confirm this finding and establish an experimental system for our studies, we compared 22-month-old mice with 6-month-old mice, which are considered fully grown young adults. The cohorts of 6- and 22-month-old mice were matched for total body weight (35.4 ± 1.0 and 35.7 ± 1.8 g, respectively; *p* = 0.86). However, the combined weight of the largest muscle groups in the hind limb and forelimb (quadriceps femoris and triceps brachii, respectively) was significantly reduced in the 22-month-old mice (by 18 ± 3%; Fig. 1*A*), indicating age-related muscle loss. We performed histological analyses of the quadriceps, a functionally important muscle group that is known to be severely affected by age-related muscle atrophy in humans (20). In both 6- and 22-month old mice, the quadriceps was primarily composed of type IIb fibers with the remainder composed of type IIx fibers (82 ± 2% type IIb fibers and 18 ± 2% type IIx fibers in 6-month-old mice *versus* 84 ± 2% type IIb fibers and 16 ± 2% type IIx fibers in 22-month-old mice). The relative amounts of IIb and IIx fibers were not affected by age (*p* = 0.49), and the size of IIx fibers was not affected by age (Fig. 1*C*). Importantly, however, the size of IIb fibers was significantly reduced in 22-month-old mice (Fig. 1, *B–D*). Furthermore, 22-month-old mice exhibited a significant reduction in grip strength (decreased by 12 ± 4%; Fig. 1*E*) and a significant reduction in specific force (*i.e.* muscle quality; decreased by 36 ± 11%; Fig. 1*F*). These age-related reductions in muscle mass, type IIb fiber size, strength, and muscle quality are indicative of age-related muscle atrophy as it has been described in both mice and humans (1, 2, 20).

**FIGURE 2.**
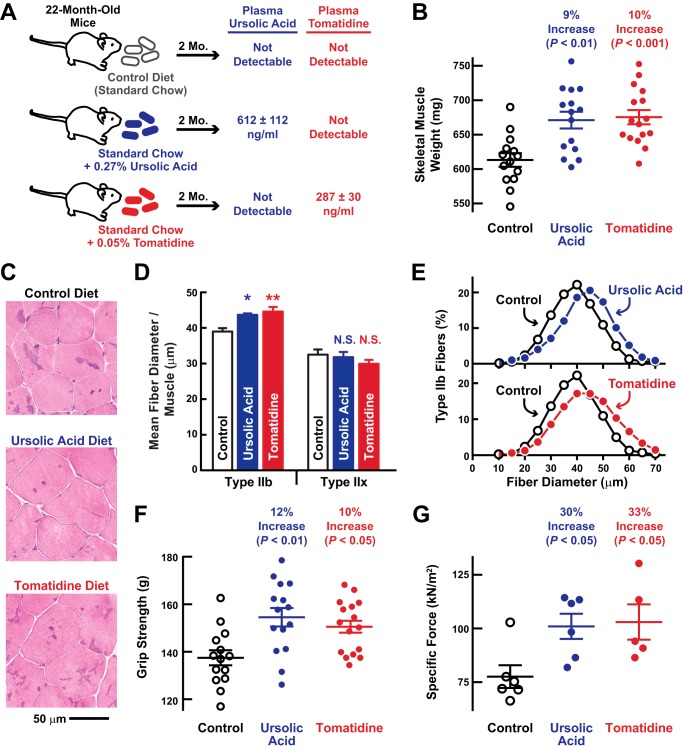
**Ursolic acid and tomatidine reduce age-related skeletal muscle weakness and atrophy.** *A–G*, weight-matched cohorts of 22-month-old male C57BL/6 mice were provided *ad libitum* access to standard chow (“control”) or standard chow supplemented with 0.27% ursolic acid or 0.05% tomatidine for 2 months before the analyses described below. *A*, steady-state plasma levels of ursolic acid and tomatidine. Data are means ± S.E. from three mice per cohort. *B*, combined weight of bilateral quadriceps femoris and triceps brachii muscles. Each data point represents one mouse, and *horizontal bars* denote means ± S.E. *C*, representative H&E images of quadriceps cross-sections. *D*, mean diameters of quadriceps muscle fibers. Data are averages of the mean diameters of quadriceps type IIb and IIx fibers from four mice per cohort ±S.E. *, *p* < 0.05; **, *p* < 0.01. *N.S.* denotes *p* > 0.05. *E*, size distribution of all type IIb fibers from *D. F*, *in vivo* forelimb grip strength. Each data point represents the mean of five measurements from one mouse, and *horizontal bars* denote the average of the means ± S.E. *G*, *ex vivo* specific tetanic force generated by the extensor digitorum longus muscle. Each data point represents one mouse, and *horizontal bars* denote means ± S.E. In *B*, *D*, *F*, and *G*, *p* values were determined with a one-way ANOVA with Dunnett's multiple comparison test. *kN*, kilonewtons.

To test the hypothesis that ursolic acid and tomatidine might reduce age-related skeletal muscle weakness and atrophy, we provided weight-matched cohorts of 22-month-old mice *ad libitum* access to diets lacking or containing either 0.27% ursolic acid or 0.05% tomatidine for 2 months (Fig. 2*A*). We previously found that these doses of ursolic acid and tomatidine stimulate skeletal muscle hypertrophy and increase strength and specific force in young adult mice (10, 11, 21). The diets containing ursolic acid and tomatidine were well tolerated by 22-month-old mice. Altogether in these studies, we observed 32 mice on each diet (control, ursolic acid, or tomatidine), and by the end of the 2-month treatment period, four control mice had died of natural causes, one ursolic acid-treated mouse died, and no tomatidine-treated mice died. As expected, the diet containing ursolic acid generated measurable plasma levels of ursolic acid, and the diet containing tomatidine generated measurable plasma levels of tomatidine (Fig. 2*A*).

**FIGURE 3.**
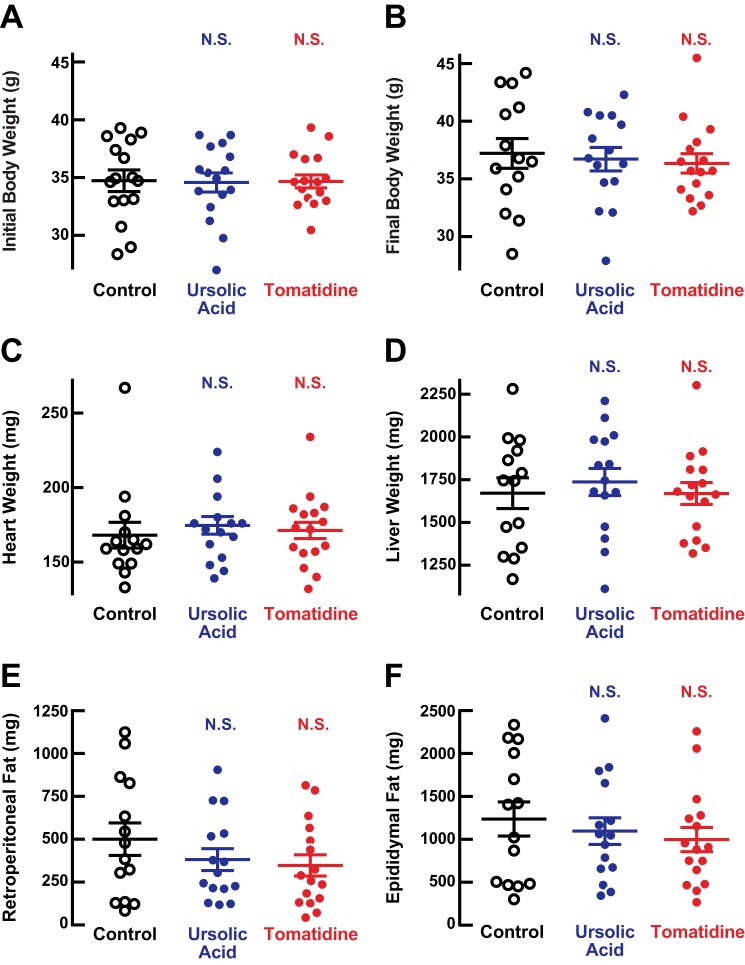
**In aged mice, ursolic acid and tomatidine do not alter total body weight or the weights of heart, liver, or fat pads.** *A–F*, weight-matched cohorts of 22-month-old male C57BL/6 mice were provided *ad libitum* access to standard chow (control) or standard chow supplemented with 0.27% ursolic acid or 0.05% tomatidine for 2 months. Each data point represents one mouse, and *horizontal bars* denote means ± S.E. *A*, initial total body weights. *B*, final total body weights. *C*, heart weights. *D*, liver weights. *E*, combined weights of bilateral retroperitoneal fat pads. *F*, combined weights of bilateral epididymal fat pads. *p* values were determined with a one-way ANOVA with Dunnett's multiple comparison test. *N.S.* denotes *p* > 0.05.

We found that ursolic acid and tomatidine significantly increased skeletal muscle weight by 9 ± 2 and 10 ± 2%, respectively (Fig. 2*B*). In addition, both compounds significantly increased the size of type IIb muscle fibers in the quadriceps without increasing the size of type IIx fibers (Fig. 2, *C–E*). Ursolic acid and tomatidine did not alter the relative amount of IIb and IIx fibers in the quadriceps (there were 97 ± 2, 96 ± 2, and 96 ± 1% IIb fibers in control, ursolic acid-treated, and tomatidine-treated quadriceps, respectively; all *p* > 0.05). In addition to reducing atrophy of type IIb fibers, ursolic acid and tomatidine significantly increased grip strength (by 12 ± 3 and 10 ± 2%, respectively; Fig. 2*F*) and significantly increased specific force (by 30 ± 8 and 33 ± 11%, respectively; Fig. 2*G*). In contrast to their effects on skeletal muscle, ursolic acid and tomatidine did not significantly alter total body weight or the weights of heart, liver, or fat pads (Fig. 3, *A–F*). Taken together, these data indicate that ursolic acid and tomatidine reduced age-related muscle weakness and atrophy.

##### In Aged Skeletal Muscle, Ursolic Acid and Tomatidine Generate Similar mRNA Expression Signatures, Which Are Composed of Hundreds of Small Positive and Negative Changes in mRNA Levels

Age-related muscle atrophy is a complex and slowly progressive process that remains poorly understood at the molecular level. Because ursolic acid and tomatidine reduced age-related muscle weakness and atrophy, we reasoned that they might provide insight into the molecular pathogenesis of age-related muscle loss. To test this hypothesis, we performed additional analyses of quadriceps muscles of 22-month-old mice that had consumed diets lacking or containing ursolic acid or tomatidine for 2 months (as in Figs. 2 and 3).

**FIGURE 4.**
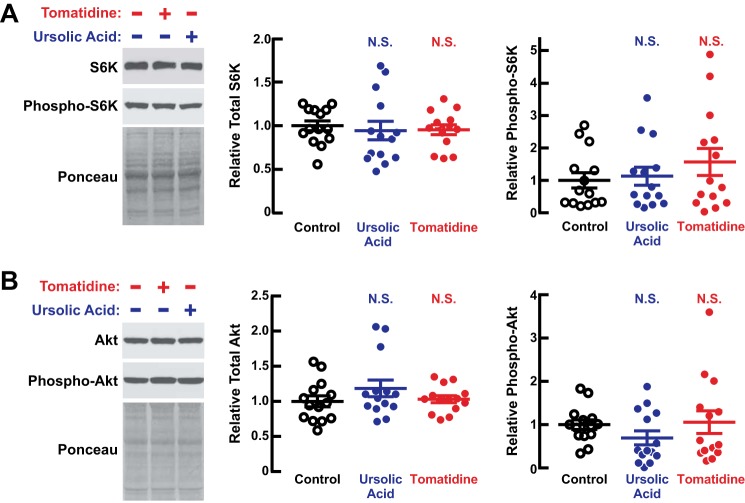
**The steady-state level of Akt/mTORC1 signaling in aged skeletal muscle is not altered after 2 months of treatment with ursolic acid or tomatidine.** Data are from the quadriceps muscles of 22-month-old C57BL/6 mice treated for 2 months with control diet, ursolic acid diet, or tomatidine diet as in Figs. 2 and 3. *A*, quadriceps protein extracts were subjected to immunoblot analysis with anti-S6K and anti-phospho-S6K antibodies. *Left*, representative immunoblots and Ponceau S stains. *Middle*, quantification of total S6K. *Right*, quantification of the normalized ratio of phospho-S6K to total S6K in each extract. *B*, quadriceps protein extracts were subjected to immunoblot analysis with anti-Akt and anti-phospho-Akt antibodies. *Left*, representative immunoblots and Ponceau S stains. *Middle*, quantification of total Akt. *Right*, quantification of the normalized ratio of phospho-Akt to total Akt in each extract. *Horizontal bars* denote means ± S.E. *p* values were determined with a one-way ANOVA and Dunnett's multiple comparison test. *N.S.* denotes *p* > 0.05.

In young adult mice, ursolic acid and tomatidine promote muscle hypertrophy by increasing activity of the protein kinase mTORC1 (10, 11, 21). We therefore tested the possibility that one or both of the compounds might increase phosphorylation (activity) of S6 kinase, a key downstream target of mTORC1, in aged skeletal muscle. However, we found that ursolic acid and tomatidine did not increase the steady-state level of S6 kinase phosphorylation in aged muscle (Fig. 4*A*). Similarly, we did not detect any change in phosphorylation (activity) of Akt (Fig. 4*B*), an anabolic protein kinase that mediates the effect of ursolic acid on mTORC1 (10, 21). Although we cannot rule out the possibility that ursolic acid and/or tomatidine may have stimulated Akt and/or mTORC1 at some point during the 2-month treatment period, it was clear that Akt/mTORC1 signaling was not significantly increased at the end of the treatment period. The absence of sustained mTORC1 activation in ursolic acid- and tomatidine-treated muscles is perhaps not surprising because sustained activation of mTORC1 does not inhibit age-related muscle atrophy but rather appears to promote it (22). Furthermore, a small molecule inhibitor of mTORC1, rapamycin, does not accentuate or reduce age-related muscle atrophy (23). These results led us to investigate other potential mechanisms.

**FIGURE 5.**
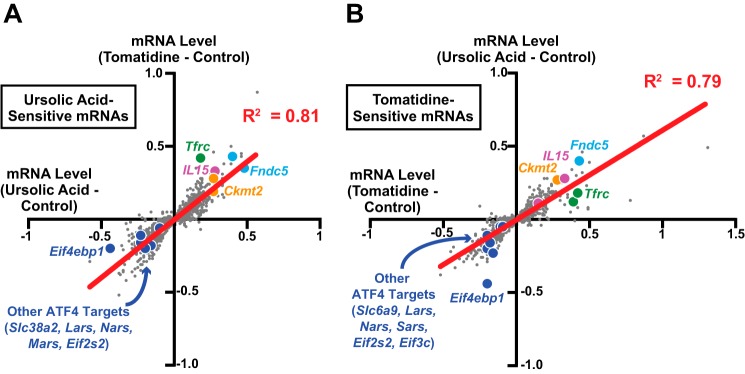
**The mRNA expression signatures of ursolic acid and tomatidine in aged skeletal muscle are highly similar and include repression of ATF4-dependent mRNAs.** *A* and *B*, data are from quadriceps muscles of 22-month-old C57BL/6 mice in Fig. 2 that were treated for 2 months with control diet, ursolic acid diet, or tomatidine diet. Quadriceps mRNA was analyzed with Mouse Ref-8 version 2.0 BeadChip arrays, which contain >25,000 probes to >19,000 mRNA transcripts. *n* = 5–6 arrays per condition. Effects of ursolic acid and tomatidine on individual probe signals were determined by normalizing log_2_ signals from ursolic acid-treated mice and tomatidine-treated mice, respectively, to log_2_ signals from control mice. Statistical significance was arbitrarily defined as *p* ≤ 0.025 by a two-tailed unpaired *t* test. *A*, scatter plot showing the effects of ursolic acid and tomatidine on the 1289 mRNA probes whose signals were significantly altered by ursolic acid. Some mRNAs of interest are highlighted. *B*, scatter plot showing the effects of tomatidine and ursolic acid on the 1031 mRNA probes whose signals were significantly altered by tomatidine. Some mRNAs of interest are highlighted.

We originally identified ursolic acid and tomatidine as small molecules whose collective effects on gene expression in human cell lines are roughly opposite to the changes in skeletal muscle gene expression that occur during muscle atrophy (10, 11). This suggested the hypothesis that ursolic acid and tomatidine might reverse at least some changes in skeletal muscle gene expression that promote age-related muscle atrophy. To begin to test this idea, we used genome-wide mRNA expression arrays to assess mRNA expression in quadriceps muscles of 22-month-old mice that had consumed diets lacking or containing ursolic acid or tomatidine for 2 months. Using *p* ≤ 0.025 by two-tailed unpaired *t* test as an arbitrary cutoff for statistical significance, we found that ursolic acid affected the signals from 1289 mRNA probes (about 5% of the >25,000 probes measured on the arrays; supplemental Table 1), and tomatidine affected signals from 1031 mRNA probes (supplemental Table 2). The magnitude of these changes in mRNA levels was small with few changes greater than 2-fold. Remarkably, there was a strong correlation between the effects of ursolic acid and tomatidine: in most cases, if one compound was judged to have a statistically significant effect on a particular mRNA, then the other compound affected that mRNA in the same general direction (up or down) even if the effect was not judged to be statistically significant (Fig. 5, *A* and *B*). When the data were subjected to an FDR analysis, mRNA probe signals that met a cutoff of FDR ≤ 0.1 (supplemental Table 3) constituted a subset of the mRNA probe signals that met the less stringent cutoff of *p* ≤ 0.025, and there remained a strong correlation between the effects of ursolic acid and tomatidine (*R*^2^ = 0.92; *p* < 0.0001). Thus, the antiatrophic effects of ursolic acid and tomatidine in aged skeletal muscle were associated with numerous small positive and negative changes in skeletal muscle mRNA expression, and there was a surprisingly high concordance between the effects of the two compounds.

##### Gene Set Enrichment Analysis Identifies ATF4 as a Transcription Factor Whose Activity Is Reduced by Ursolic Acid and Tomatidine in Aged Skeletal Muscle

**FIGURE 6.**
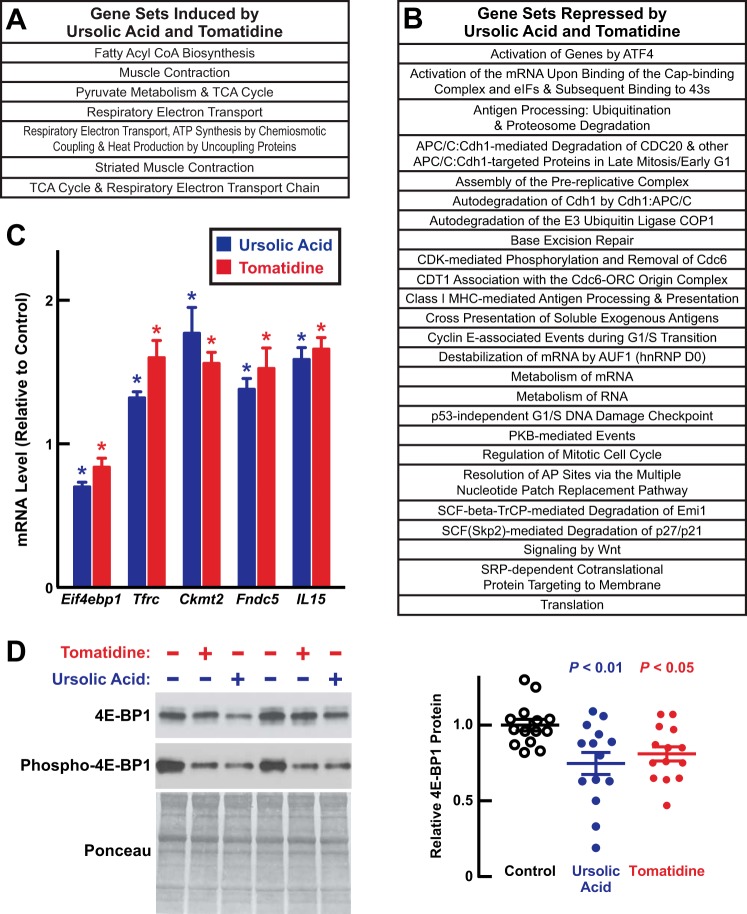
**Ursolic acid and tomatidine significantly induce or repress numerous gene sets in aged skeletal muscle, including repression of the gene set activation of genes by ATF4.** *A* and *B*, array data from Fig. 5 were subjected to gene set enrichment analysis. Statistical significance was defined as *p* < 0.05 and FDR < 0.25. *A*, reactome gene sets that were significantly induced in the quadriceps of both ursolic acid-treated mice and tomatidine-treated mice relative to control mice. *B*, reactome gene sets that were significantly repressed in the quadriceps of both ursolic acid-treated mice and tomatidine-treated mice relative to control mice. *C* and *D*, data are from quadriceps muscles of 22-month-old C57BL/6 mice in Fig. 2 that were treated for 2 months with control diet, ursolic acid diet, or tomatidine diet. *C*, levels of the indicated transcripts were assessed with qPCR. Data are means ± S.E. from five mice per condition. *D*, quadriceps protein extracts were subjected to immunoblot analysis with anti-4E-BP1 or anti-phospho-4E-BP1 antibodies. *Left*, representative immunoblots. Membranes were stained with Ponceau S to confirm equal loading. *Right*, quantification of total 4E-BP1. Each data point represents one mouse, and *horizontal bars* denote means ± S.E. In *C* and *D*, *p* values were determined with a one-way ANOVA with Dunnett's multiple comparison test. *, *p* ≤ 0.05. *TCA*, tricarboxylic acid; *hnRNP*, heterogeneous nuclear ribonucleoprotein; *CDK*, cyclin-dependent kinase; *APC/C*, *anaphase-promoting complex/cyclosome; SRP,* signal recognition particle; *ORC*, origin recognition complex; *AP*, *apurinic/apyrimidinic.*

Because ursolic acid and tomatidine had similar effects on skeletal muscle mRNA levels, we used gene set enrichment analysis (24) to search for basic cellular processes that might be stimulated or inhibited by ursolic acid and tomatidine in aged skeletal muscle. We found that both compounds induced seven gene sets that promote either muscle contraction or mitochondrial bioenergetics (Fig. 6*A*). Consistent with the gene set enrichment analysis results, mRNA expression arrays and qPCR analysis showed that ursolic acid and tomatidine increased mRNAs encoding proteins involved in muscle growth and/or mitochondrial bioenergetics (*e.g.* transferrin receptor and mitochondrial creatine kinase, encoded by *Tfrc* and *Ckmt2*, respectively) as well as mRNAs encoding the contraction-induced transcripts *IL-15* and *Fndc5* (Figs. 5, *A* and *B*, and 6*C*).

We also used gene set enrichment analysis to search for gene sets that were repressed by ursolic acid and tomatidine. Altogether, we found that both compounds repressed 25 gene sets that represent a diverse range of cellular functions, including “activation of genes by ATF4” (Fig. 6*B*). ATF4 is a bZIP transcription factor subunit that mediates a variety of cellular stress responses (25, 26). The potential role of ATF4 in age-related muscle atrophy had not been investigated.

All of the gene sets influenced by ursolic acid and tomatidine (Fig. 6, *A* and *B*) represent potentially important processes in age-related muscle atrophy. However, we chose to focus on the activation of genes by ATF4 set for three reasons. First, we wished to identify a cellular process that actively drives age-related muscle atrophy, and we reasoned that such a process would most likely be found within the gene sets that were repressed by ursolic acid and tomatidine. Second, ATF4 is known to be required for some, but not all, forms of acute muscle atrophy (14, 15, 18, 27). Although endogenous ATF4 protein cannot be reliably detected in skeletal muscle (presumably due to its low abundance, very short half-life, and the absence of high quality antibodies), targeted knock-out of the *Atf4* gene in skeletal muscle fibers partially prevents acute muscle atrophy during fasting and limb immobilization (14, 15, 18). Conversely, forced expression of ATF4 is sufficient to induce atrophy of skeletal muscle fibers *in vivo* and skeletal myotubes *in vitro* (14, 15).

Third, a variety of impairments in skeletal muscle protein synthesis have been implicated as important contributing factors in age-related muscle atrophy (2, 28, 29), and we hypothesized that ATF4-mediated gene expression might have a capacity to reduce protein synthesis in skeletal muscle. This hypothesis was based on the finding that ursolic acid and tomatidine repressed several mRNAs that encode proteins that can potentially influence global protein synthesis, including *Eif4ebp1*, *Eif2s2*, and *Eif3c* (which encode proteins that regulate translation initiation); *Slc6a9* and *Slc38a2* (which encode amino acid transporters); and *Lars*, *Nars*, *Mars*, and *Sars* (which encode aminoacyl-tRNA synthetases) (Fig. 5, *A* and *B*). All of these transcripts arise from established ATF4 target genes (30), and *Eif4ebp1* in particular encodes a well established inhibitor of global protein synthesis, 4E-BP1. qPCR analysis confirmed that ursolic acid and tomatidine reduced *Eif4ebp1* mRNA in aged skeletal muscle (Fig. 6*C*), and importantly, the reduction in *Eif4ebp1* mRNA was accompanied by a reduction in 4E-BP1 protein (Fig. 6*D*). Ursolic acid and tomatidine did not alter the level of mTORC1-mediated 4E-BP1 phosphorylation (inactivation), consistent with the finding that the compounds did not alter mTORC1-mediated S6 kinase phosphorylation. These data suggested that ursolic acid and tomatidine reduced the level of active (unphosphorylated) 4E-BP1 and perhaps other inhibitors of protein synthesis. Together, these considerations led us to focus on ATF4 as a potential negative regulator of skeletal muscle protein synthesis and a potentially important driver of age-related skeletal muscle atrophy.

##### ATF4 Reduces Skeletal Muscle Protein Synthesis

**FIGURE 7.**
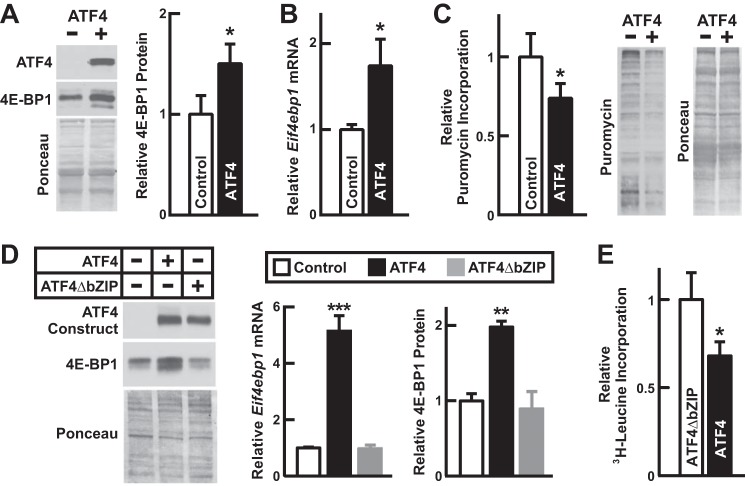
**ATF4 reduces skeletal muscle protein synthesis.** *A–C*, we used 2-month-old male C57BL/6 mice and transfected one tibialis anterior muscle with 20 μg of empty plasmid (pcDNA3; control) and the contralateral tibialis anterior with 20 μg of pcDNA3 encoding FLAG-tagged ATF4 (p-ATF4-FLAG). *A* and *B*, bilateral tibialis anterior muscles were harvested 3 days post-transfection. *A*, tibialis anterior protein extracts were subjected to immunoblot analysis using anti-FLAG and anti-4E-BP1 antibodies. *Left*, representative immunoblots and Ponceau S stains. *Right*, quantification of 4E-BP1. Data are means ± S.E. from nine mice. *B*, qPCR analysis of *Eif4ebp1* mRNA. Data are means ± S.E. from four mice. *C*, skeletal muscle protein synthesis was assessed by administering intraperitoneal injections of puromycin 3 days post-transfection, harvesting bilateral tibialis anterior muscles 30 min later, and then subjecting tibialis anterior protein extracts to immunoblot analysis with an anti-puromycin antibody. *Left*, quantification of incorporated puromycin. Data are means ± S.E. from five mice. *Right*, representative immunoblot and Ponceau S stain. *D*, fully differentiated C2C12 myotubes were infected for 48 h with recombinant adenoviruses expressing GFP alone (control), GFP plus FLAG-tagged ATF4, or GFP plus a full-length, transcriptionally inactive FLAG-tagged ATF4 construct (ATF4ΔbZIP). *Left*, representative anti-FLAG and anti-4E-BP1 immunoblots and Ponceau S stains. *Middle*, qPCR analysis of *Eif4ebp1* mRNA. Data are means ± S.E. from six replicates/condition. *Right*, quantification of 4E-BP1. Data are means ± S.E. from four replicates/condition. *E*, myotube protein synthesis was assessed by infecting C2C12 myotubes for 48 h with recombinant adenoviruses expressing GFP plus ATF4ΔbZIP or GFP plus FLAG-tagged ATF4 and then measuring [^3^H]leucine incorporation into total cellular protein. Data are means ± S.E. from five experiments. In *A–E*, *p* values were determined with *t* tests (*A–C* and *E*) or a one-way ANOVA with Dunnett's multiple comparison test (*D*). *, *p* < 0.05; **, *p* < 0.01; ***, *p* < 0.001. *Error bars* denote means ± S.E.

To begin to investigate the potential effect of ATF4 on skeletal muscle protein synthesis, we overexpressed ATF4 in skeletal muscle fibers of young adult (2-month-old) mice (Fig. 7*A*). As expected, ATF4 increased *Eif4ebp1* mRNA (Fig. 7*B*) and 4E-BP1 protein (Fig. 7*A*). To determine the effect of ATF4 on muscle protein synthesis, we used the SUnSET method, which assesses *in vivo* incorporation of low dose puromycin into elongating polypeptide chains (16). We found that ATF4 significantly reduced the incorporation of puromycin into total skeletal muscle protein (Fig. 7*C*), indicating a reduction in global protein synthesis. Thus, increased ATF4 expression is sufficient to reduce protein synthesis in skeletal muscle.

As a complementary system for investigating the effect of increased ATF4 expression, we used a recombinant adenovirus to overexpress ATF4 in cultured C2C12 myotubes, an *in vitro* model of skeletal muscle (Fig. 7*D*). In this system, we compared wild-type ATF4 with a transcriptionally inactive ATF4 construct that contains seven point mutations in the bZIP domain (ATF4ΔbZIP; Fig. 7*D*). Similar to its effects in skeletal muscle *in vivo*, ATF4 increased *Eif4ebp1* mRNA and 4E-BP1 protein in cultured skeletal myotubes (Fig. 7*D*). In contrast, the transcriptionally inactive ATF4ΔbZIP construct did not increase either *Eif4ebp1* mRNA or 4E-BP1 protein (Fig. 7*D*). We then assessed global protein synthesis with a traditional metabolic labeling method and found that ATF4 significantly decreased protein synthesis relative to ATF4ΔbZIP (Fig. 7*E*). These data suggest that ATF4-dependent gene expression leads to a reduction in skeletal muscle protein synthesis.

##### Targeted Reduction in Skeletal Muscle ATF4 Expression Increases Protein Synthesis in Aged Skeletal Muscle and Reduces Age-related Muscle Weakness and Atrophy

To determine whether a reduction in ATF4 expression might increase protein synthesis in aged skeletal muscle, we studied 22-month-old ATF4 mKO mice. These mice carry two copies of a floxed *Atf4* allele as well as the muscle creatine kinase (*MCK*)*-Cre* transgene, which excises the floxed *Atf4* alleles in skeletal muscle fibers and heart (14, 18, 31, 32). We compared the ATF4 mKO mice with littermate control mice, which also carry two copies of the floxed *Atf4* allele but lack the *MCK-Cre* transgene. Importantly, ATF4 mKO mice undergo normal muscle development and are phenotypically normal under basal conditions as young adults (14, 18).

**FIGURE 8.**
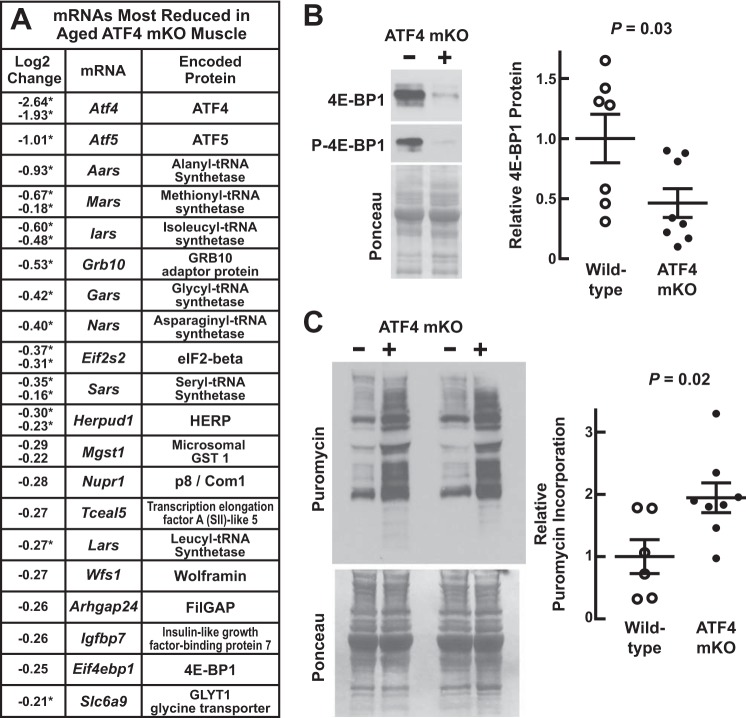
**Targeted reduction of ATF4 expression in aged skeletal muscle increases muscle protein synthesis.** *A–C*, we compared weight-matched cohorts of 22-month-old male ATF4 mKO mice (*i.e. ATF4*(*L*/*L*);*MCK-Cre*(*Tg*/*0*) mice) and *ATF4*(*L*/*L*);*MCK-Cre*(*0*/*0*) littermate control mice. *A*, quadriceps mRNA was analyzed with Mouse Ref-8 version 2.0 BeadChip arrays. *n* = 8 arrays per genotype. Effects of ATF4 mKO on individual probe signals were determined by normalizing log_2_ signals from ATF4 mKO mice to log_2_ signals from control mice. Statistical significance was arbitrarily defined as *p* ≤ 0.025. The data show the 20 mRNAs whose levels were most reduced in ATF4 mKO muscle relative to control muscle; some mRNAs have two log_2_ changes because they were represented by two probes on the array. *Asterisks* indicate probes that were also considered significantly reduced by FDR analysis (*i.e.* FDR ≤ 0.1). *B*, quadriceps protein extracts were subjected to immunoblot analysis with anti-4E-BP1 and anti-phospho-4E-BP1. *Left*, representative immunoblots and Ponceau S stains. *Right*, quantification of total 4E-BP1. Each data point represents one mouse, and *horizontal bars* denote means ± S.E. *C*, skeletal muscle protein synthesis was assessed by administering intraperitoneal injections of puromycin, harvesting the quadriceps muscles 30 min later, and then subjecting quadriceps protein extracts to immunoblot analysis with an anti-puromycin antibody. *Left*, representative immunoblot and Ponceau S stain. *Right*, quantification of incorporated puromycin. Each data point represents one mouse, and *horizontal bars* denote means ± S.E. In *B* and *C*, *p* values were determined with *t* tests. *FilGAP*, *filamin A-binding GTPase-activating protein.*

Fig. 8*A* shows the 20 mRNA transcripts whose levels were most reduced in 22-month old ATF4 mKO quadriceps relative to quadriceps from 22-month old littermate controls (the complete list is shown in supplemental Tables 4 and 5). As expected, the largest reduction was in *Atf4* mRNA (Fig. 8*A*), and this was accompanied by modest reductions in multiple mRNAs that encode regulators of protein synthesis, including *Eif4ebp1* (Fig. 8*A*). The reduction in *Eif4ebp1* mRNA was associated with a reduction in 4E-BP1 protein (Fig. 8*B*). Aged ATF4 mKO muscles did not contain a reduced level of *Gadd45a* mRNA, which mediates acute atrophic effects of ATF4 during fasting and limb immobilization (14) but is also known to be controlled by an ATF4-independent mechanism (27). We used the SUnSET method to assess quadriceps protein synthesis in 22-month-old control and ATF4 mKO mice. Relative to control muscles, ATF4 mKO muscles had a significantly higher level of puromycin incorporation into total muscle protein (Fig. 8*C*). Thus, a targeted reduction in skeletal muscle ATF4 expression increases global protein synthesis in aged skeletal muscle.

**FIGURE 9.**
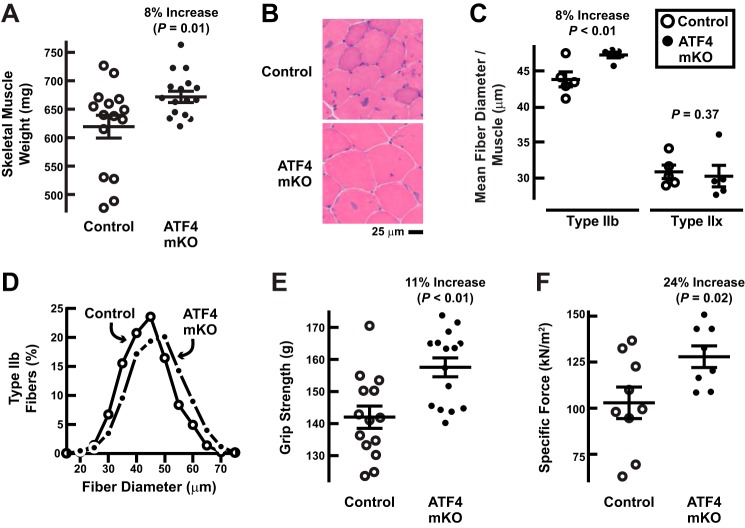
**Targeted reduction of skeletal muscle ATF4 expression reduces age-related muscle weakness and atrophy.** *A–F*, we compared weight-matched cohorts of 22-month-old male ATF4 mKO mice and littermate control mice. *A*, combined weight of bilateral quadriceps femoris and triceps brachii muscles. Each data point represents one mouse, and *horizontal bars* denote means ± S.E. *B*, representative H&E images of quadriceps cross-sections. *C*, average diameters of type IIb and IIx fibers in the quadriceps. Each data point represents the mean diameters of type IIb or IIx fibers from one mouse, and *horizontal bars* denote average of the means ± S.E. *D*, size distribution of all type IIb fibers from *C. E*, *in vivo* forelimb grip strength. Each data point represents the mean of five measurements from one mouse, and *horizontal bars* denote the average of the means ± S.E. *F*, *ex vivo* specific tetanic force generated by the extensor digitorum longus muscle. Each data point represents one mouse, and *horizontal bars* denote means ± S.E. In *A*, *C*, *E*, and *F*, *p* values were determined with *t* tests. *kN*, kilonewtons.

We next asked whether ATF4 mKO mice might be resistant to age-related muscle weakness and atrophy. At 22 months of age, there was no difference in total body weight between ATF4 mKO mice and littermate controls (total body weights were 39.3 ± 1.4 g in control mice and 38.1 ± 2.1 g in ATF4 mKO mice; *p* = 0.45). However, ATF4 mKO mice possessed significantly larger skeletal muscles (increased by 8 ± 2%; Fig. 9*A*). The weights of heart, liver, and fat pads were not significantly changed; in control and ATF4 mKO mice, heart weights were 162 ± 6 and 161 ± 4 mg, respectively (*p* = 0.46); liver weights were 1755 ± 99 and 1694 ± 71 mg, respectively (*p* = 0.31); epididymal fat pad weights were 1414 ± 219 and 1402 ± 183 mg, respectively (*p* = 0.48); and retroperitoneal fat pad weights were 612 ± 130 and 533 ± 71 mg, respectively (*p* = 0.30).

We performed histological analyses of the quadriceps muscles of 22-month-old control and ATF4 mKO mice and found that ATF4 mKO muscles had significantly larger type IIb fibers (Fig. 9, *B–D*). The relative amounts of IIb and IIx fibers were unchanged (87 ± 3 and 92 ± 2% type IIb fibers and 13 ± 3 and 8 ± 2% type IIx fibers in control and ATF4 mKO mice, respectively; *p* = 0.13). In addition to increasing muscle mass and IIb muscle fiber size, muscle-specific *Atf4* gene deletion significantly increased *in vivo* grip strength (by 11 ± 2%; Fig. 9*E*) and *ex vivo* specific force (by 24 ± 6%; Fig. 9*F*). Taken together, these data indicate that a targeted reduction of skeletal muscle ATF4 activity reduces age-related muscle weakness and atrophy, similar to ursolic acid and tomatidine.

##### ATF4 Is Required for a Small Subset of the Gene Expression Changes That Are Generated by Ursolic Acid and Tomatidine in Aged Skeletal Muscle

**FIGURE 10.**
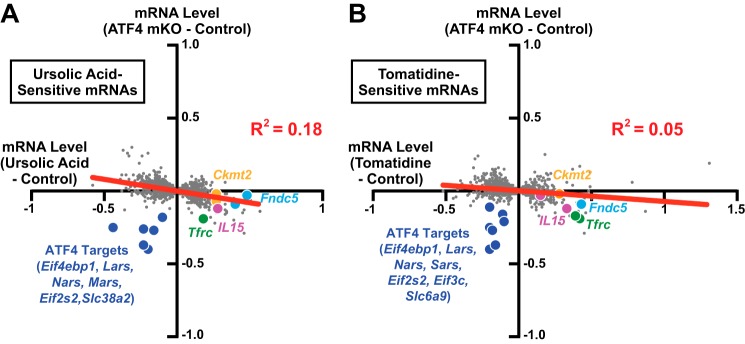
**ATF4 is required for only a small portion of the gene expression changes that are generated by ursolic acid and tomatidine in aged skeletal muscle.** *A* and *B*, data are from the microarray studies described in Figs. 5 and 8. *A*, scatter plot showing the effects of *Atf4* gene deletion on quadriceps mRNAs whose levels were significantly altered by ursolic acid. *B*, scatter plot showing the effects of *Atf4* gene deletion on quadriceps mRNAs whose levels were significantly altered by tomatidine.

Because *Atf4* gene deletion, ursolic acid, and tomatidine had similar morphological and functional effects in aged skeletal muscle, we asked whether *Atf4* gene deletion might generate the same changes in skeletal muscle gene expression that were generated by ursolic acid and tomatidine. Fig. 10, *A* and *B*, show the effects of *Atf4* gene deletion on mRNAs whose levels were significantly altered by ursolic acid or tomatidine in aged skeletal muscle. As expected, *Atf4* gene deletion reduced levels of multiple ATF4-dependent mRNAs (*e.g. Eif4ebp1*, *Lars*, *Nars*, *Mars*, *Sars*, *Slc6a9*, *Slc38a2*, *Eif2s2*, and *Eif3c*), similar to ursolic acid or tomatidine (Fig. 10, *A* and *B*, and supplemental Tables 6 and 7). However, in contrast to ursolic acid and tomatidine, *Atf4* gene deletion did not increase mRNAs involved in muscle contraction and mitochondrial bioenergetics, and the overall correlation between the effects of *Atf4* gene deletion and the effects of the compounds was poor (Fig. 10, *A* and *B*). Thus, ATF4 mediates a small subset of the effects that ursolic acid and tomatidine exert on skeletal muscle gene expression, and inhibiting this small subset of ATF4-dependent effects appears to be sufficient to reduce age-related skeletal muscle weakness and atrophy.

## Discussion

One of the goals of the current study was to identify a small molecule that reduces age-related skeletal muscle weakness and/or atrophy. To this end, we tested two small molecules, ursolic acid and tomatidine, in elderly mice. Both ursolic acid and tomatidine were known to reduce acute muscle atrophy in young adult mice during fasting and muscle disuse, but their effects in aging were unknown. The current results indicate that 2 months of dietary supplementation with either ursolic acid or tomatidine significantly reduces age-related deficits in skeletal muscle mass, strength, and quality. These results identify two novel small molecule inhibitors of age-related skeletal muscle weakness and atrophy.

To better understand mechanisms of age-related muscle atrophy and weakness, we performed an unbiased analysis of the effects of ursolic acid and tomatidine on gene expression in aged skeletal muscle. We found that ursolic acid and tomatidine generate hundreds of small positive and negative changes in mRNA levels in aged skeletal muscle. Moreover and despite their structural differences, ursolic acid and tomatidine generate remarkably similar mRNA expression signatures, which, based on pathway analysis, appear to impact a broad range of cellular processes. Although these data do not speak to the direct or acute effects of ursolic acid and tomatidine in aged skeletal muscle, they do suggest that the end result of a 2-month treatment with either compound is a subtle reprogramming of skeletal muscle gene expression. Furthermore, these subtle changes in gene expression are accompanied by a reduction in age-related muscle weakness and atrophy and may reflect a moderation of at least some of the molecular effects of skeletal muscle aging.

All of the genes that are induced or repressed by ursolic acid and tomatidine in aged skeletal muscle could potentially contribute to age-related muscle weakness and atrophy. In the current study, we focused our attention on a small subset of genes that are repressed by ursolic acid and tomatidine. This particular subset of ursolic acid- and tomatidine-sensitive genes is known to be directly activated by ATF4, and our data indicate that a targeted reduction in skeletal muscle ATF4 expression not only represses this subset of genes but also reduces age-related muscle weakness and atrophy. To our knowledge, ATF4 is the first example of a skeletal muscle protein in mammals that is required for the decline of skeletal muscle strength, quality, and mass during aging. The discovery of ATF4 as a key mediator of age-related muscle weakness and atrophy provides an important foundation for future investigations into the molecular basis of skeletal muscle aging.

As a transcription factor, ATF4 could potentially influence many cellular processes in aged skeletal muscle. In the current study, we investigated the effects of ATF4 on one cellular process, skeletal muscle protein synthesis, which is known to be impaired in association with age-related muscle weakness and atrophy. Our data indicate that a targeted reduction in ATF4 expression is sufficient to increase protein synthesis in aged skeletal muscle, and conversely, forced expression of ATF4 is sufficient to decrease protein synthesis in young adult skeletal muscle. These results, coupled with the effects of ATF4 on muscle strength, quality, and mass, suggest that ATF4 may promote age-related muscle weakness and atrophy at least in part by reducing skeletal muscle protein synthesis. The mechanism by which ATF4 reduces protein synthesis is likely complex because ATF4 regulates multiple genes that could influence protein synthesis at multiple levels, including amino acid transport, aminoacyl-tRNA availability, and mRNA translation. One potentially relevant ATF4 target gene is *Eif4ebp1*, which encodes 4E-BP1, a well established repressor of cap-dependent mRNA translation.

ATF4 also promotes acute muscle atrophy during fasting (14, 15), and many of the mRNAs that are positively regulated by ATF4 in aged skeletal muscle are also positively regulated by ATF4 in young adult skeletal muscle during fasting. Examples include *Eif4ebp1*, *Eif2s2*, *Lars*, *Nars*, *Mars*, *Iars*, *Aars*, and *Gars* (14, 15). However, there also appear to be some important differences in the transcripts that are regulated by ATF4 during fasting and aging. The most notable example is *Gadd45a*, which is induced by ATF4 during fasting and contributes to muscle atrophy during fasting (14, 15). Although aging is known to increase skeletal muscle *Gadd45a* expression (33–37), the current data indicate that ATF4 is not required for *Gadd45a* expression in aged skeletal muscle. This finding suggests that lowering *Gadd45a* mRNA is not absolutely required to reduce age-related muscle weakness and atrophy. It also suggests that *Gadd45a* expression in aged skeletal muscle may be mediated by HDAC4, which is known to induce *Gadd45a* mRNA in an ATF4-independent manner (27).

By identifying ATF4 as an indirect or direct target of ursolic acid and tomatidine, the current study identifies two small molecules that blunt ATF4-dependent effects in aged skeletal muscle. Understanding how ursolic acid and tomatidine reduce skeletal muscle ATF4 activity is an important and challenging area for future investigation. The current data do not indicate whether ursolic acid and tomatidine reduce ATF4 activity by the same or different mechanisms, and there are many potential mechanisms given the multitude of pathways, often interconnected, that regulate ATF4 activity. Importantly, a reduction in ATF4 activity cannot explain all of the effects of ursolic acid and tomatidine in skeletal muscle. For example, this mechanism cannot explain how ursolic acid and tomatidine stimulate muscle hypertrophy because a targeted reduction in skeletal muscle ATF4 expression does not induce muscle hypertrophy (14, 15). In addition, ATF4 accounts for only a small portion of the mRNAs that are regulated by ursolic acid and tomatidine in aged skeletal muscle, and it seems improbable that all of the ATF4-independent effects of ursolic acid and tomatidine are functionally irrelevant. Rather, we speculate that age-related muscle atrophy proceeds via a highly complex molecular signaling network with several critical and vulnerable nodes. One of these nodes is represented by ATF4, but others likely exist and may be affected by ursolic acid and tomatidine in ways that we do not yet understand.

The progrowth effects of ursolic acid and tomatidine in young adult skeletal muscle (*i.e.* hypertrophy and recovery from atrophy) are associated with activation of mTORC1, a well established mediator of muscle growth (10, 11).^3^ In addition, the mTORC1 inhibitor rapamycin prevents ursolic acid- and tomatidine-mediated hypertrophy in cultured myotubes (11).^3^ Interestingly, however, the current data indicate that ursolic acid and tomatidine reduce age-related muscle atrophy and weakness without eliciting a sustained increase in mTORC1 activity. Because of our study design, we cannot rule out the possibility that ursolic acid and tomatidine may transiently stimulate mTORC1 at an early treatment time point in aged skeletal muscle. However, given the potentially deleterious effect of sustained mTORC1 activity in skeletal muscle aging (22) and the finding that ursolic acid and tomatidine improve muscle strength, quality, and mass in aged muscle, it is perhaps not surprising that ursolic acid and tomatidine do not generate a sustained increase in mTORC1 activity in aged muscle. Taken together, the current data suggest that, whereas ursolic acid and tomatidine promote muscle growth via an mTORC1-dependent mechanism, they reduce age-related muscle weakness and atrophy via a mechanism that is at least somewhat less dependent on mTORC1 and more dependent on inhibition of atrophy mediators, such as ATF4.

In light of the current results, ursolic acid and tomatidine represent potential agents and/or lead compounds for medical treatment of age-related muscle weakness and atrophy. In addition, because ursolic acid and tomatidine naturally occur in food, they could potentially comprise or contribute to nutritional products aimed at preserving strength and muscle mass during aging. If ursolic acid- and tomatidine-based approaches are found to be safe and effective in humans, they could possibly be used alone, together, or in combination with physical therapy and other nutritional and pharmaceutical approaches. Similar to other complex chronic conditions such as type 2 diabetes, dyslipidemia, and hypertension, age-related muscle weakness and atrophy may ultimately require a repertoire of modalities that can be used alone or in combination depending on the clinical circumstances. In summary, the current study provides new insight into the molecular pathogenesis of age-related skeletal muscle weakness and atrophy and elucidates new potential approaches for preventing and treating the loss of strength and muscle mass during aging.

## Author Contributions

S. M. E. and C. M. A. conceived and coordinated the study, designed the experiments, and wrote the paper. S. M. E., M. C. D., S. A. B., J. M. D., D. J. M., D. K. F., K. S. B., V. A. L., D. K. M., and J. J. T. performed the experiments and contributed to the preparation of the manuscript and figures. All authors reviewed the results and approved the final version of the manuscript.

## Supplementary Material

Supplemental Data

## Footnotes

3 S. M. Ebert, M. C. Dyle, S. A. Bullard, J. M. Dierdorff, D. J. Murry, D. K. Fox, K. S. Bongers, V. A. Lira, D. K. Meyerholz, J. J. Talley, and C. M. Adams, unpublished results.

## Footnotes

2 The abbreviations used are:

ATF4: activating transcription factor 4
S6K: *S6 kinase*
4E-BP1: eukaryotic translational initiation factor 4E-binding protein 1
SUnSET: surface sensing of translation
mKO: *muscle-specific knock-out*
*qPCR*: quantitative real time RT-PCR
ANOVA: analysis of variance
FDR: false discovery rate
bZIP: *basic leucine zipper*
*MCK*: muscle creatine kinase.
