# Supplementary material for: Identification and Small Molecule Inhibition of an Activating Transcription Factor 4 (ATF4)-dependent Pathway to Age-related Skeletal Muscle Weakness and Atrophy
Source: J Biol Chem. 2015 Sep 3;290(42):25497–511. doi: 10.1074/jbc.M115.681445 (PMC4646196; doi:10.1074/jbc.M115.681445)
Supplement: Supplemental Data [file supp_290_42_25497__index.html]

Identification and Small Molecule Inhibition of an ATF4-dependent Pathway to Age-related Skeletal Muscle Weakness and Atrophy — Identification and Small Molecule Inhibition of an Activating Transcription Factor 4 (ATF4)-dependent Pathway to Age-related Skeletal Muscle Weakness and Atrophy — Mechanism and Small Molecule Inhibition of Sarcopenia — Supplemental Data 

# Identification and Small Molecule Inhibition of an Activating Transcription Factor 4 (ATF4)-dependent Pathway to Age-related Skeletal Muscle Weakness and Atrophy

## Supplemental Data

- Supplemental tables (.xlsx, 439 KB) - Supplemental data from microarray studies
